# Supplementary material for: Expression profiles of the immune genes CD4, CD8β, IFNγ, IL-4, IL-6 and IL-10 in mitogen-stimulated koala lymphocytes (Phascolarctos cinereus) by qRT-PCR
Source: PeerJ. 2014 Mar 4;2:e280. doi: 10.7717/peerj.280 (PMC3961157; doi:10.7717/peerj.280)
Supplement: Supplemental Information 1 [file peerj-02-280-s001.pdf]

Supplementary Material A: Multiple sequence alignment of marsupial CD4, CD8β, IFNγ, IL-4, IL-6, IL-10, and GAPDH. Boxed sequences are qPCR primers. GenBank accession numbers are listed in Table 1.

CD4

```
Phci CD4   GTGTTCAAGGTGACAGCCAACCCAAGTGACTCTGTGTTCTCTGGAACCAACGTGATTTTAACCTTGCACGGCTCTTCCAA  : 80
Saha CD4   ----------....G..........A...T.A.A....T.......AG...A...C......A..A..TA..C.C....G  : 452
Maeu CD4   ...................C...........A....A.............T....C........................  : 712
Modo CD4   ....................T........G......C.......G.A........C......T......A..C..C....  : 714

Phci CD4   CCTTCCTGCACTCAAGGTGGAATGGAGTGGTCCAGGAGACAAAAGTAAAAGGATCCTGAGTCAAGACAAGAAAACTCTGA  : 160
Saha CD4   ........G.T...............A............T....................................T...  : 532
Maeu CD4   ..........T.T..............G...........T.........CA........A................T...  : 792
Modo CD4   ........A......AA.A......CA..........A.T.C...................TC.A..........CT...  : 794

Phci CD4   AGTTGGTGCAAATAGAGACCAGCGAAGGGGGTGTGTGGGAYTGTACCGTCTCTGTGAATGACAAAGCCCTGAAATTGAGC  : 240
Saha CD4   .C..AA.C..T..G...C...AA.....A...A.T...T.....GATA............G....A..............  : 612
Maeu CD4   .C......A....G.G.C.A.A....AC....C.C..........TT..........G...A...A...........G..  : 872
Modo CD4   .C...C.C...G.G..TT.TGAG..G.A...A.AA...AG...C..T.....CA.A....G....AG...T...C.....  : 874

Phci CD4   ATCACAGTCACAGTGTTTGGTTTCACATCGTCCTCCCAGACCTTCTACATGATGACAGGCAAACCTGCCAAATTCTCCTT  : 320
Saha CD4   ....A.........A..............AC.A.........A....TGG.............A...T.C..........  : 692
Maeu CD4   ....A........CA............AAA..T..T...........T.C....GTG......G...T..G.........  : 952
Modo CD4   .AA.G.........ACA........G.CACC...A....CG..A...T.A...AG........GA....G.....AA...  : 954

Phci CD4   CCCTCTGAATTTAAACGAACAGGATCTGAACAGGGAACATCCAAATGGAGAACTGAGGTGGCAGGTGGAAGGTGCTGCCT  : 400
Saha CD4   ...............T..G.....G.......A...GA.......A......T.C.CA................T.....  : 772
Maeu CD4   ...............T..C..A..G.....T........G.....................A.CA.......CA...T..  : 1032
Modo CD4   ......A......GG................C..ATGGTG................CT...C...G......G.......  : 1034

Phci CD4   CTCCCCCCCGGTCGGCCAAGTTTTCATGGAAGGGTGACTCTTTGACTCTAGAAAAGGGGGCTCCAAATTTCAGTCGTGAT  : 480
Saha CD4   ......AA.A.C.A.......A...........A.A.TAA......G.CCA....T...AT...........AAGCG..G  : 852
Maeu CD4   ....A.---A.GT..T................A.......C.......G..G..CA.AC......CC.....A.......  : 1109
Modo CD4   ...AAA---...T....................A......C........C...G.CAA.--.TT..G...A..CT..CCA  : 1109

Phci CD4   ---CCTAAGTTCCCACTCACCATCACTCTTCATCCTGTCTTGCCTCAGCATGCTGGCTCAGGAGTCTT  : 525
Saha CD4   AAGT....T...G..A....AT........TCC...........C.......................  : 920
Maeu CD4   ---..C..............G.........TCCT......C....TCT....................  : 1174
Modo CD4   AAGG.AG.----...A....TT.G.G...CTCC..A...C...TC..C....................  : 1173
```

CD8β

```
Phci CD8β 1   ACCCACCAAAAAGACCACTCCCAAGAGGAGAGTATGCAATACAAC---CCCAAAGGTCACTCAACAGAATGGTTCCCTCT  : 77
Phci CD8β 2   .............................................---................................  : 77
Saha CD8β     .........G........C....G....C.GA............AACC.T..G.T...........A......A...A..  : 517
Maeu CD8β     ..................C....G..A....CCG........G.AACA.T..G.A...................T.T...  : 554
Modo CD8β     ..................CA.A.......A.A.G...CCC.A..AATT....GGT.C.........AG.....C.TT...  : 515

Phci CD8β 1   GCAGCGCCTTCACCCTAAGCCTGCTGGTGGGATGTGCTGTGGTTTTGCTGATCTCCATCATTGTGATCATCCGATTGAAT  : 157
Phci CD8β 2   .........................................A......................................  : 157
Saha CD8β     .T.A.AT.C..C.T..C.....C........G......AC.........A.....TC.A....................C  : 597
Maeu CD8β     ....T...C..C................................C....C...TC.C.A...............A.....  : 634
Modo CD8β     ....T.T.C.GCT...C..................ATCC..A..............CA..........C.....A....C  : 595

Phci CD8β 1   TATTTATGGAATGTAGCTCGGCACCACTTTGTGAAACAGCTTCAAAGATGAGTACCCCTTCCCACTCAACCCTCCCCCCA  : 237
Phci CD8β 2   ................................................................................  : 237
Saha CD8β     ............C.......T..T....................C...........G..C.AT..C.CT.----------  : 667
Maeu CD8β     ..C..G.....................G.C......T.......G.........T....CT....C.T..T.C.------  : 708
Modo CD8β     ..C......C..C.G.....C..T................................AA---------------------.  : 653

Phci CD8β 1   AAAGATAAAGTTCTTCAAATAGCCTGCCGAAGAAATGCACTGGATTTTATATAAGCAGAATTGGAGGAACTGAGGCATGA  : 317
Phci CD8β 2   ................................................................................  : 317
Saha CD8β     -..A.......................T..........G.........GC..............-.A....AGA....CT  : 745
Maeu CD8β     -..A..........C......A....TTA..............G.....C................A.......A...C.  : 787
Modo CD8β     ..CA.AG.....T.C......A.....T........A.......C...GC.G.............AA..TGAG.....C.  : 733

Phci CD8β 1   GAACAGCAATGA--ACTTTTGAAATTTTCCTAAGCCACTGGGGT  : 359
Phci CD8β 2   ............--..............................  : 359
Saha CD8β     ...T.A......TG.ACC........C....G....-...----  : 784
Maeu CD8β     ...T........--..C...........................  : 829
Modo CD8β     ...T...G....--.ACA....C....................C  : 775
```

IFNγ

```
Phci IFNγ  CAAGCTNCNTCTTAGCATCCTGGCTTTGTGTTATGTTGGGTT------------ATTCTCAAGCTACTATAGCAGAAGAC : 68
Saha IFNγ  ......A.C............TT........C..TC..A...CTTCTGGCTGTCT........T..ACC..AG....... : 80
Maeu IFNγ  ......A.C..............T....CC............------------........--....G..AG-...... : 65
Modo IFNγ  ......T.G....................A.C..T.......ATTCTGGCTATGT......G.........A..AC...T : 80

Phci IFNγ  CTAGGAACTCTTATGGAATACTTTCAGAATGGAAGTACATCATCTGATATATCTGATAATGG---GACTCTTTTCTTGAA : 145
Saha IFNγ  A.GCA.......CACA.C......---....C..C..A..---....CG....A...GG.A.---TT.......TA..G. : 154
Maeu IFNγ  ...AA..T.........C......---.........-...--..............A.....---............... : 139
Modo IFNγ  ..G.C........AC..C......---....C....GT..---.....G..G.....GG.A.TCG.C........ACA.. : 157

Phci IFNγ  CATGATGGATCGTTGGAAAGAGG---ATGGTGACAAGAAAATCCTAATGAGCCAAATTGTCACAGTTTATTTCAAAATCT : 222
Saha IFNγ  .......A.GAC...........GAA.CT..............T..........TG.....G.................. : 234
Maeu IFNγ  .........C..C..........---.CA..........G...A........-........................... : 215
Modo IFNγ  .......A.A.............---.CA.........G...T...........T...A..................... : 234

Phci IFNγ  TTGAAATCTTCAAGAACAACACCGTCATCAAAAAAAGTGTGGAGAATATCAAAGAGGATATGATCATGAAATTCTTCACT : 302
Saha IFNγ  .C...........A......T..A.TG......G....A.....C......G.....................A..TC.. : 314
Maeu IFNγ  .C...........A.........AC......G.....CC.......C..............................C.. : 295
Modo IFNγ  ..........G...G.....T..A.....C.......CC...TA...........A...........A.........CA. : 314

Phci IFNγ  AACAACACTGCCAGCAAAGTGAATGACTTTGAATCTGTCATCAACACTCAGGTGAATGACCTCAGAGTCCAAAAGAAAGC : 382
Saha IFNγ  ...............TCT...G..........GG.AC.......T..C................A........G...... : 394
Maeu IFNγ  ...........T.....................G.G...............----------------------------- : 346
Modo IFNγ  .........TT..A..TG.CA..A..TA.....GA.C......................TT...A........G...... : 394

Phci IFNγ  TATATTTGAACTAGCCCTTATCATGAATGACCTCTCAAACAAACCTCATCTAAGAAAGAGAAAAAGAAGACAGAACANAA : 462
Saha IFNγ  ...G..........T.TA.G.TT.CCG.A........CCG.............C.GG.....G................. : 474
Modo IFNγ  ..............ATGAA......C.A.........TC............C............................ : 474

Phci IFNγ  GCCA : 466
Saha IFNγ  .... : 478
Modo IFNγ  .... : 478
```

IL-4

```
Phci IL-4  GTCTCACATTCCAACTGATACCAACTTTGTTCTGCCTGTTAACATGTACCAGTGATGTTGTCCAGGGACAGAGAAGGACC : 80
Maeu IL-4  .....................................C..........................A.............T. : 149
Modo Il-4  ....................T............A.A.A.........C.........C.TC.T.T....C..A..A..GG : 84

Phci IL-4  TGCCAACCCTCTCTCAGAGAGATCGTCCACATGGCAAACTCTCTTACTATGAAAAAGTTTCCCTGCTTTGAGATGGAAGT : 160
Maeu IL-4  .....GA.....G.T.A.......A......CT.............T.G............................... : 229
Modo Il-4  ......AGT...G.T.........A..G..TAT.TG....T...C...CC...C......T......C..C......... : 164

Phci IL-4  GCCGGACATCTTTGAGGATACAGAGAATTCCAGTACTCCGAAGCTGTTTCCTGAGGAGTCATCATGCTCTCAGCTGGTCA : 240
Maeu IL-4  ......................A................A...T...CG....T...A...................... : 309
Modo Il-4  ......................A.........AC.....A...T.A---......A.T...................... : 241

Phci IL-4  TGACAATGAATGACTTTGAAACATTATGCAGAGCTGTCACTGTGCTCCAACAGGTCTCCAACAGCTGCCCATTCCCTAAG : 320
Maeu IL-4  .......................C....................................G.......A..C........ : 389
Modo Il-4  ......G.............................C...G.........G...C.....G..AT...A..A...GCC.. : 321

Phci IL-4  CTAAGCAACATGCTTAGGAACCTAATGCACTTAGTGAATCAGACCAAATGTCCTGTGAATGAAACCAAGATTACTAAATT : 400
Maeu IL-4  A...A......................T.............................................T...... : 469
Modo Il-4  .....A.T...A.AA..........A.TTT.............T...T.........................T...... : 401

Phci IL-4  GCAAGATTTCCTGAGCA : 417
Maeu IL-4  ................. : 486
Modo Il-4  ................. : 418
```

IL-6

```
          Intron 3
Phci IL-6  AACACCTGTTTGGCTTTTAGCAAAACCATTGAAGTGATGTCTCTGTTTGTCCAGATGGAAAACAAGGCAGCTGGATTTTT  : 80
Modo IL-6  ........................GT..........G......A..................GC.....A.....C....
Saha IL-6  ....................TG...T..C.......G.....T..C................T............C....

Phci IL-6  CCAAGTCCATAGGGCCATTGGGAGGAGAAATGAGATGATATTACAGGAGAAGCACTTTGATTTTGTTAGAGGGGTAAAGG  : 160
Modo IL-6  ....C....C...AT............-.............CCT.A...T...CT...A....G...CA.......GG..
Saha IL-6  ....C............C..............G.........GT..A....A..T...A..................G.A

Phci IL-6  AAA-----GGGAAAAGGAGCCATACAAGCTCTGCTTTAAAAGGTCAATTTTAATTATTTTTATATAATACTTTTTTCCTA  : 235
Modo IL-6  ...AAAAT..A...G.AG..A..C....A.G...........AC........T..TA.....C.C...G.-.C..A....
Saha IL-6  ...G----...G.....G..A...T...T........G....AC.T......-A.TA.........C.--.....C..C.

                                                        ▶Exon 4
Phci IL-6  TTGGTAACCATAATTTTTCTCTT----TTTCACGTAAGGAGACATGCTTGCCAAGGATTGTGAGTGGTCTTCAGGAGTTT  : 311
Modo IL-6  ....C...T..............TTTA......A................AT..A...CA.C...........A..C...
Saha IL-6  .C......T.G.........A..TTT----...A.............C..AT..ACC.CA.C...AAA..G.....A...

Phci IL-6  GACACGTATCTTCGGTACATGGAAACAGAGATGAAGGATAA------TAAGTTACAATACCTAAGAATGGGTACAGTTCA  : 385
Modo IL-6  ....TA.T....AAC...........T......G.......------C.G...T...AC.....AGC..A.C..TAC...
Saha IL-6  ...GG....T.C.A..T........AT...C....A..A..GAAAAGC.G.A.CG..GCA....A....TTC..GAC...

                              ▶Intron 4
Phci IL-6  GCTCATCCACAAACTGAAGCTCTTGGTGAGTTTCCCTTTGC  : 426
Modo IL-6  AT.AGC.A....CT.....AC.G..................
Saha IL-6  A..AGC.G.GTCT.....AAAA...................
```

IL-10

```
Phci IL-10  ACATGCTCCGAGAACTTCGAGCAGCCTTCAGCAACGTGAAGATCTACTTTCAAACCAGAGACAAGCTCGAAACC---AAA  : 77
Trvu IL-10  .................................G........CA...........G...........G......---...  : 250
Saha IL-10  T.T.A...............A...G....GAGG.A......GGT..........G..A....G.AT.GATTT.TGTG.G.  : 183
Modo IL10   .............T...............G.T..A...................AG.........T.G......---.G.  : 180

Phci IL-10  TTGATAGACAAATCTTTGCTGGAAGAATTAAAGAGTTACCTGGGCTGCCAGGCCTTGTCAGAGATGATTCAGTTTTACCT  : 157
Trvu IL-10  .....................................................................A..........  : 330
Saha IL-10  ...T....TG.GC.............C..C................................C......AC.........  : 263
Modo IL10   C.A.......G...............C................................CC.......CA..........  : 260

Phci IL-10  GGAGGAAGTGATGCCTCAGGCAGAGAAGAATGAAGTGGACATCAAAGAGAACGTGGGCTCTTTAGGCGAGAAGCTGAAGG  : 237
Trvu IL-10  .................G.......G....C...C....TG........G..........C.....A.....AT......  : 410
Saha IL-10  ...A..T.....A....G.....---........AC...G.....GA.C.G.....T...C...AAA..C.......T..  : 340
Modo IL10   ....A..................---.......G.A..G.G..........A........C..G..A..C..AT.AC..A  : 337

Phci IL-10  CACTGAGATTGAGGCTCAAACGCTGTCACAGATTCCTGCCCTGTGAAGATAGGAGCAGAGTTGTCAAGCAAGTCAAGAGC  : 317
Trvu IL-10  ........C..........G..A..............C...........C.AC.....G................G..A.  : 490
Saha IL-10  GG......AG..CA.......AA.................T.......TC.AA..T.CT.C....C..A..A........  : 420
Modo IL10   TT......C.....................................G..CGA.....AG..G..T..T.GC......GA.  : 417

Phci IL-10  ACCTATGAGAAGCTCCAGGAACAAGGAGTCTACA--------------------------------  : 351
Trvu IL-10  ......A..G........................AAGCAATGGGGGATTTTGACATATTCATCGGC  : 556
Saha IL-10  .........C.A...A.T.GTA.T.....TCT..AAGCAATGGGGGAGTTTAATATATTCATCAAC  : 486
Modo IL10   ..................................AAGCTATGGGGGATTTTGACATATTCATCAAC  : 483
```

GAPDH

```
Phci GAPDH  AGTCCACTGGCGTGTTTACCACCATGGAAAAGGCTGGGGCTCACTTGAAGGGTGGAGCCAAGAGGGTCATCATCTCTGCC  : 80
Saha GAPDH  ............................G.............................T...C.A..............A  : 400
Maeu GAPDH  -----------------------------...................................................  : 51
Modo GAPDH  ..............................................................C.C.....T.........  : 201

Phci GAPDH  CCTTCTGCTGATGCCCCAATGTTCGTGATGGGAGTGAACCATGAGAAATATGATAATTCCCTTAAGATTGTCAGTAACGC  : 160
Saha GAPDH  .....C.................T........G....................C....................C..T..  : 480
Maeu GAPDH  .............................................................................T..  : 131
Modo GAPDH  ..C.............................G........C........C.....C.....C.....C........T..  : 281

Phci GAPDH  TTCCTGCACTACCAATTGCTTGGCCCCCCTGGCCAAGGTCATTCATGACAACTTTGGCATTGTGGAAGGACTCATGACTA  : 240
Saha GAPDH  C..............C........T...T....N...........C................A...............C.  : 560
Maeu GAPDH  ...............C........T...T...................................................  : 211
Modo GAPDH  C..............C....................A........C........C.......................C.  : 361

Phci GAPDH  CAGTCCATGCCATTACTGCTACCCAGAAGACAGTAGATGGCCCCTCTGGTAAGCTGTGGCGTGATGGGCGTGGTGCTTCC  : 320
Saha GAPDH  ....T........C........T...........C..C...........C...........................G..  : 640
Maeu GAPDH  .............................................................................G..  : 291
Modo GAPDH  ................C................................C.......................G...G..  : 441

Phci GAPDH  CAAAACATCATCCCTGCTTCCACTGGTGCTGCTAAAGCTGTAGGCAAGGTCATACCTGAGCTGAATGGGAAGCTCACAGG  : 400
Saha GAPDH  ..G....................C...........G..C..........................C..A..A........  : 720
Maeu GAPDH  ...................................G............................................  : 371
Modo GAPDH  .....T.................C.....C..C..G.....G..............C........C..............  : 521

Phci GAPDH  CATGGCCTTCCGTGTTCCTACTCCCAATGTATCTGTTGTGGATCTGACCTGCCGTCTGGAAAAACCTGCCAAATATGATG  : 480
Saha GAPDH  ......T.....C..............C..G.......................C.....G.....C.............  : 800
Maeu GAPDH  ......................................................C.....G...................  : 451
Modo GAPDH  ..................C........C..G..C..G...........T.....C.....G...G..........C....  : 601

Phci GAPDH  ATATCAAGAAAGTGGTGAAGGAAGCATCAGAAGGACCCTTGAAGGGCATCTTGGGCTACACAGAGGACC-----------  : 549
Saha GAPDH  .C..................C.....G....T......A..............................AGGTTGTATCC  : 880
Maeu GAPDH  ....................C.G........G.....................................AGGTTGTGTCC  : 531
Modo GAPDH  .C........G.........C....G.....G...G....A..A.........................AGGTTGTATCC  : 681
```
